# Phenotypic and genotypic characterization of erythromycin-resistant *Staphylococcus aureus* isolated from bovine subclinical mastitis in Egypt

**DOI:** 10.14202/vetworld.2023.1562-1571

**Published:** 2023-07-31

**Authors:** Khaled A. Abd El-Razik, Amany A. Arafa, Ehab A. Fouad, Ashraf H. Soror, Abeer M. Abdalhamed, Magdy Elgioushy

**Affiliations:** 1 Department of Animal Reproduction, National Research Centre, Dokki, Egypt; 2 Department of Microbiology and Immunology, National Research Centre, Dokki, Egypt; 3 Department of Zoonosis, National Research Centre, Dokki, Egypt; 4 Department of Parasitology and Animal Diseases, National Research Centre, Dokki, Egypt; 5 Department of Animal Medicine, Faculty of Veterinary Medicine, Aswan University, Egypt

**Keywords:** antimicrobial resistance, bovines, Egypt, erythromycin resistance, genetic diversity, *Staphylococcus aureus*, subclinical mastitis

## Abstract

**Background and Aim:**

Subclinical mastitis (SCM) caused by erythromycin-resistant *Staphylococcus aureus* is a significant disease in lactating animals. Therefore, it is crucial to understand the genetic factors contributing to erythromycin resistance in *S. aureus*. This study aimed to estimate the prevalence of *S. aureus* in milk from subclinical mastitic cattle and buffaloes and tank milk samples as identified by probe-based real-time polymerase chain reaction (PCR) and the genotypic assessment of macrolide and erythromycin resistance profiles, as well as to analyze the phylogenetic relatedness of our local isolates of *S. aureus*.

**Materials and Methods:**

In total, 285 milk samples were analyzed using the California mastitis test to detect SCM. Milk samples were cultured on different specific *Staphylococcus* media. The presence of *S. aureus* was confirmed by Gram staining, the catalase and coagulase tests, the detection of hemolytic activity, DNase agar testing, and biofilm activity in Congo red medium. The genotypic identification of *S. aureus* (*nuc*) was performed. The determinants of erythromycin (*erm*A, *erm*B, *erm*C, and *erm*T) and macrolide resistance (*msr*A) were screened in all isolates. DNA sequencing of our local isolates of *S. aureus* was used to analyze their phylogenetic relatedness. Moreover, histopathological examination of tissue specimens of mammary gland was performed.

**Results:**

The *S. aureus* positivity rates were 36.4%, 48.8%, and 63.6% in cattle, buffalo, and bulk tank milk, respectively. Probe-based real-time PCR molecularly confirmed all 62 *S. aureus* isolates. Thirty-one isolates were subjected to PCR to create profiles of their genotypic erythromycin resistance. *erm*A, *erm*B, *erm*C, and *erm*T were present in 5 (8%), 26 (41.9%), 18 (29%), and 15 (24.1%) *S. aureus* isolates, respectively. Moreover, *msr*A was found in three (4.8%) strains. Eight PCR products were produced using standard PCR for DNA sequencing. Multiple sequence alignment, phylogenetic tree construction, and analysis of *nuc* in *S. aureus* revealed a high degree of homology (100%) with *S. aureus* strains isolated from milk in cases of bovine mastitis in India and Kenya. Histological analysis of udder tissues revealed extensive aggregation of mononuclear inflammatory cells in the interstitial connective tissue, primarily lymphocytes, and macrophages.

**Conclusion:**

This study showed a high prevalence of erythromycin resistance in *S. aureus* isolates. This information is vital for controlling mastitis and the spread of resistance genes between bacterial strains and hosts. Moreover, the probe-based real-time PCR approach is helpful for the rapid screening of *S. aureus* isolates and the consequent efficient treatment and control of *S. aureus* mastitis.

## Introduction

Bovine mastitis is the most prevalent developing infectious disease globally, affecting the amount and quality of milk [1, 2]. Pathogens, the environment, and animals jointly contribute to the development of this inflammatory illness. Although subclinical mastitis (SCM) lacks overt symptoms, clinical mastitis (CM) can be identified by obvious abnormalities in milk and swelling or discomfort of the udder [3]. Depending on the host and pathogen interactions, mastitis can present as either CM or SCM [4]. Subclinical mastitis is the most prevalent form in all dairy animals [5, 6]. The California mastitis test (CMT), which determines the presence of cellular nuclear protein in milk samples, is an affordable, straightforward, and rapid mastitis screening test. As inflammatory cells are the most prevalent cells observed in mastitic milk, the CMT accurately represents the somatic cell count (SCC), and it is a valid indicator of the extent of infection. The process is sufficiently easy for milking employees and producers to learn, and the test is suitable for the cow-side examination of udder health [7]. For dairy animals, mastitis is an international issue that affects animal health and decreases milk output and quality, resulting in financial losses [8, 9]. Many pathogenic, opportunistic, and spoilage microorganisms best flourish in lactating cow milk, thereby affecting the pathophysiology of mastitis [10, 11]. Such microbes include *Staphylococcus aureus*, *Streptococcus* spp., *Klebsiella pneumoniae*, *Escherichia coli*, and other less frequent pathogens such as *Pseudomonas aeruginosa*, *Mycoplasma* spp., and *Mycobacterium* spp. [12, 13]. As staphylococci cause contagious infection, unsanitary farming practices contribute to its spread among herds. In multidrug-resistant (MDR) strains in particular, this disease can cause persistent infections that are challenging to treat with conventional therapy, causing enormous financial losses in the dairy business [14, 15]. Another major source of food-borne disease is *S. aureus* isolates from nursing animals, with bulk and raw milk products playing a key role in bacterial transmission to humans [16]. Moreover, *S. aureus* is responsible for at least 10% of food-borne illnesses linked to dairy products [17]. Real-time polymerase chain reaction (PCR) is a reliable diagnostic technique for improving bacterial identification and it offers benefits for increasing throughput, permitting completely objective interpretation of results, and quantifying the amount of microbes in milk samples [18, 19].

The rapid detection of *S. aureus* is a critical issue for treatment and the implementation of infection control measures. In addition, real-time PCR has 100% sensitivity and specificity for these targets compared to culture and conventional techniques [20]. Similarly as all procedures, real-time PCR assays require thorough validation to avoid producing false-positive or false-negative results [21]. The clinical importance of detecting bacterial nucleic acids rather than living cells should be considered. Real-time PCR is limited to the detection of bacterial species for which a test kit has been created [22]. Nonetheless, real-time PCR remains a promising molecular technique for detecting MDR pathogens produced because of the excessive use of antimicrobials in veterinary care and agriculture [15, 23, 24]. Erythromycin is an antibiotic used as an alternative to cephalosporin, penicillin, and other beta-lactams for gram-positive microorganisms, and it has been used for many illnesses for a long time [25, 26]. However, erythromycin use has led to the synthesis of methylase encoded by the *erm* genes, leading to erythromycin resistance in *S. aureus*. Erythromycin resistance in bovine mastitis isolates is reportedly caused by *erm*A, *erm*B, and *erm*C [27]. Bovine mastitis and people have both produced strains of erythromycin-resistant *S. aureus* [2]. Because *S. aureus* strains are resistant to numerous medicines, many therapeutic approaches for controlling mastitis have been ineffective [28]. Although *S. aureus* in dairy cattle has long been known cause serious harm, no effective preventive measures or treatments have been suggested. This is attributable to the lack of knowledge regarding the relationship between the bacteria and the host and the rapidly evolving genetic diversity of the pathogens.

This study aimed to estimate the prevalence of *S. aureus* in bovine subclinical mastitic milk samples, histopathologically examine udder tissues exhibiting mastitis, identify *S. aureus* isolates by probe-based real-time PCR, and detect the macrolides and erythromycin resistance profile genotypically. Moreover, this study analyzed the phylogenetic relatedness of our local isolates of *S. aureus* to explain the possible genetic link between other isolates.

## Materials and Methods

### Ethical approval

This study was approved (no. 231712012023) by Medical Research Ethics Committee at the National Research Centre, Egypt.

### Study period and location

This study was conducted from January to June 2022 at the Veterinary Research Institute, National Research Center, Dokki, Egypt.

### Animals and milk samples processing

Seventy-five bovine herds raised in private dairy farms located in Cairo, Giza, Kalyobia, Fayoum, and Kafr El-Sheikh, Egypt, were included in the study. Each herd consisted of 30–50 milking animals. Some farms contained more than one bulk tank. Samples were taken from each bulk tank in sterile vials from the top surface of the milk under agitation according to the recommendations of the National Mastitis Council, USA. [29]. Agitation was permitted to last for at least 10 min. The CMT was used to identify SCM and milk samples were obtained aseptically as suggested by Andrews *et al*. [30]. Positive CMT milk samples were tightly closed, refrigerated at 4°C, and transported to the laboratory, where they were cultivated on agar medium for 24 or 48 h.

Furthermore, 285 milk samples were collected from 150 dairy cattle and 135 dairy buffalo raised by smallholders in different governments in Egypt (Cairo, Giza, Kalyobia, Fayoum, and Kafr El-Sheikh). The CMT was applied to detect SCM and milk samples were aseptically collected as described by Andrews *et al*. [30]. Milk samples were tightly closed, refrigerated at 4°C, and transported to the Laboratory of Bacteriology, Department of Microbiology and Immunology, Veterinary Research Division, National Research Center for bacteriological examination as rapidly as possible.

### Bacteriological isolation and identification

To isolate *Staphylococcus* spp., milk samples of cattle and buffalo with SCM were centrifuged, and the sediment was then cultured on Baird-Parker agar, Mannitol salt agar, and blood agar 10% (Oxoid, UK) and then incubated for 1–2 days at 37°C [31]. Confirmation for *S. aureus* was achieved through gram staining, catalase and coagulase tests [32], hemolytic activity testing, DNase agar testing [33], and the biofilm activity onto Congo red medium [34].

### DNA extraction

DNA extraction from bacterial cultures was performed using a QIAamp DNA Mini kit (Qiagen, Germany, GmbH) following the manufacturer’s recommendations. Briefly, bacterial pellets were re-suspended in 200 μL of PBS and incubated with 20 μL of proteinase K and 200 μL of lysis buffer at 56°C for 10 min. After incubation, 200 μL of 100% ethanol was added to the lysate. The sample was then washed and centrifuged. Nucleic acid was eluted with 50 μL of elution buffer.

### Molecular identification using real-time PCR

The PCR TaqMan assay targeting *S. aureus* was performed using qTOWER 3G (Analytik Jena, Germany), which was used for thermocycling and fluorescence detection. Real-time PCR amplification was performed in a total volume of 20 µL containing 10 µL of 2× TOPreal TaqMan Probe quantitative PCR mixture (Cat RT600, Enzynomics) according to the manufacturer’s instructions. In addition, 0.2 µL (10 µm) of each primer, 0.4 µL (10 µm) the TaqMan probe mixture, and 2 µL of template DNA were mixed, and then distilled water was added for a final volume of 20 µL. The specific primers (*nuc2*) and probes used to identify *nuc* in *S. aureus* are listed in Table-1 [35–39]. The cycling conditions are listed in Table-2.

**Table-1:** Polymerase chain reaction primers and probes used in the study.

Gene	Sequence (5´-3´)	Amplicon size (bp)	References
*NUC1*	CTG GCA TAT GTA TGG CAA TTG TT TAT TGA CCT GAA TCA GCG TTG TCT	664 bp	[35]
*NUC2*	AAAGCGATTGATGGTGATACGGTT TGCTTTGTTTCAGGTGTATCAACCA FAM-Probe ATGTACAAAGGTCAACCAATGACATTYAGA	-------	[36]
*erm*A	TATCTTATCGTTGAGAAGGGATT CTACACTTGGCTTAGGATGAAA	139 bp	[37]
*erm*B	CTATCTGATTGTTGAAGAAGGATT GTTTACTCTTGGTTTAGGATGAAA	142 bp	[37]
*erm*C	CTTGTTGATCACGATAATTTCC ATCTTTTAGCAAACCCGTATTC	190 bp	[37]
*erm*T	ATTGGTTCAGGGAAAGGTCA GCTTGATAAAATTGGTTTTTGGA	536 bp	[38]
*msr*A	TCCAATCATTGCACAAAATC AATTCCCTCTATTTGGTGGT	163 bp	[39]

**Table-2:** Cycling conditions for the detection of genes in this study.

Gene	Init. Denat.	Denat.	Anneal.	Extention	Final ext.	Cycles
NUC1	95°C 2 min	95°C 20 s	60°C 30 s	72°C 45 s	72°C 10 min	35
NUC2 (Q-PCR)	95°C 10 min	95°C 10 s	56°C 20 s	60°C 40 s	-----	40
ermA	95°C 3 min	95°C 20 s	59°C 30 s	72°C 45 s	72°C 10 min	40
ermB	94°C 2 min	94°C 30 s	55°C 30 s	72°C 30 s	72°C 7 min	35
ermC	95°C 3 min	95°C 20 s	55°C 30 s	72°C 45 s	72°C 7 min	40
ermT	95°C 2 min	95°C 30 s	57°C 30 s	72°C 30 s	72°C 7 min	35
msrA	94°C 2 min	94°C 20 s	54°C 30 s	72°C 45 s	72°C 7 min	35

PCR=Polymerase chain reaction

### Conventional PCR to detect *S. aureus*

To identify *S. aureus* (*nuc*) and detect erythromycin resistance determinants (*erm*A, *erm*B, *erm*C, and *erm*T) and macrolide-resistant determinants (*msr*A), PCR was performed. A GS-96 gradient thermocycler (Hercuvan, Malaysia) was used for PCR in a final volume of 25 μL containing 12.5 μL of 2× COSMO PCR RED Master Mix (Cat. W1020300X, Willofort Co., UK), 0.5 μL (10 μM) of each primer (Vivantis, Malaysia), and 1 μL of target DNA. The PCR products were separated by electrophoresis on 1.5% agarose gels and then photographed and analyzed using the InGenius 3 gel documentation system (Syngene, UK). The used primers and cycling conditions are listed in Tables-1 and 2.

### DNA sequencing

The positive PCR products targeting *nuc1* were cleaned using a GeneJET™ Gel Extraction Kit (K0691, Thermo Fisher Scientific, USA) and then sequenced by MACROGEN Company (Korea) on 3730XL sequencers (Applied Biosystems, USA).

Eight PCR sequences identified in this study have been deposited in the GenBank database under the accession numbers OP821405–OP821408 and OP821409–OP821412 for *S. aureus* isolates from cattle and buffalo with SCM, respectively.

### Histopathological examination

Twelve udder tissue samples were fixed in formalin solution (10%), dehydrated in different concentrations of alcohol, embedded in paraffin, sectioned at a thickness of 4–5 μm, and stained with hematoxylin and eosin. The tissue sections were examined and photographed using a light microscope (Olympus BX 51, Tokyo, Japan) [40].

## Results

Using bulk milk samples from apparently normal animals, 18 (38.2%) and 15 (53.6%) cattle and buffaloes, respectively, exhibited SCM and reacted positively in the CMT, giving a total prevalence of SCM of 44% (Table-3).

**Table-3:** Prevalence of subclinical mastitis from cattle and buffaloes bulk tank milk using CMT.

Number	Governorate	Cattle (%)	Buffalo (%)	Total (%)
1	Cairo	1/3 (33.3)	-	1/3 (33.3)
2	Giza	2/8 (25)	3/6 (50)	5/14 (35.7)
3	Kalyobia	1/4 (25)	0/2 (0)	1/6 (16.7)
4	Fayoum	5/12 (41.7)	5/7 (71.4)	10/19 (52.6)
5	Kafr El-Sheikh	9/20 (45)	7/13 (53.8)	16/33 (48.5)
	Total	18/47 (38.2)	15/28 (53.6)	33/75 (44)

CMT=California mastitis test

**Figure-1:**
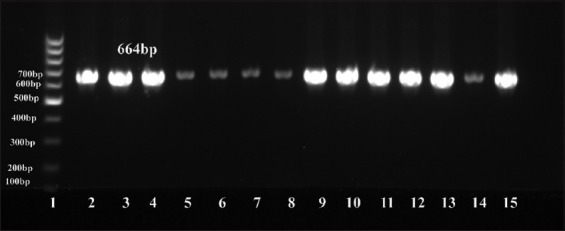
Agarose gel electrophoresis of polymerase chain reaction product amplified from *Staphylococcus aureus* *nuc* gene (664 bp). Lane 1–100 bp DNA Ladder; Lanes 2–15, representative positive samples.

Using 33 bulk tank milk samples from cattle and buffalo with SCM, 12 (66.7%) and 9 (60%) *S. aureus* isolates were identified in 18 and 15 cattle and buffalo milk samples, respectively, giving a prevalence of 63.6% (Table-4 and Figure-1).

**Table-4:** Prevalence of *Staphylococcus aureus* isolates in bulk tank milk samples from cattle and buffaloes with subclinical mastitis.

Number	Governorate	Cattle (%)	Buffalo (%)	Total (%)
1	Cairo	1/1 (100)	-	1/1 (100)
2	Giza	2/2 (100)	1/3 (33.3)	3/5 (60)
3	Kalyobia	1/1 (100)	0	1/1 (100)
4	Fayoum	4/5 (80)	2/5 (40)	6/10 (60)
5	Kafr El-Sheikh	4/9 (44.4)	6/7 (85.7)	10/16 (62.5)
	Total	12/18 (66.7)	9/15 (60)	21/33 (63.6)

In apparently normal cattle and buffaloes, 55 (36.7%) and 43 (31.9%) examined milk samples from cattle and buffaloes, respectively, exhibited SCM and reacted positively in the CMT, giving a prevalence of SCM of 34.4% (Table-5).

**Table-5:** Prevalence of SCM in dairy cattle and buffaloes at different governorates of Egypt (Smallholders).

Number	Governorate	Cattle (%)	Buffalo (%)	Total (%)
1	Cairo	4/12 (33.3)	5/15 (33.3)	9/27 (33.3)
2	Giza	11/27 (40.7)	6/25 (24)	17/52 (32.7)
3	Kalyobia	10/23 (43.5)	13/37 (35.1)	23/60 (38.3)
4	Fayoum	14/45 (31.1)	9/41 (22)	23/86 (26.7)
5	Kafr El-Sheikh	16/43 (37.2)	10/17 (58.8)	26/60 (43.3)
	Total	55/150 (36.7)	43/135 (31.9)	98/285 (34.4)

SCM = Subclinical mastitis

Using milk from subclinically mastitic cattle and buffaloes raised by smallholders, 20 (36.4%) and 21 (48.8%) *S. aureus* isolates were obtained from 55 and 43 cattle and buffaloes, respectively, giving a total prevalence of 41.8% (Table-6 and Figure-1).

**Table-6:** Prevalence of *Staphylococcus aureus* isolates in milk samples from cattle and buffaloes with subclinical mastitis (Smallholders).

Number	Governorate	Cattle (%)	Buffalo (%)	Total (%)
1	Cairo	1/4 (25)	4/5 (80)	5/9 (55.6)
2	Giza	3/11 (27.3)	6/6 (100)	9/17 (52.9)
3	Kalyobia	5/10 (50)	4/13 (30.8)	9/23 (39.1)
4	Fayoum	4/14 (28.6)	4/9 (44.4)	8/23 (34.8)
5	Kafr El-Sheikh	7/16 (43.8)	3/10 (30)	10/26 (38.5)
	Total	20/55 (36.4)	21/43 (48.8)	

### Molecular identification using real-time PCR

In total, 62 *S. aureus* isolates that were bacteriologically isolated and identified from the milk of cattle and buffaloes with SCM were subjected to molecular confirmation using probe-based real-time PCR. The probe-based real-time PCR confirmed all 62 (100%) bacteriologically identified isolates.

### Molecular identification using conventional PCR

Conventional PCR was performed using the positive probe-based reverse-transcription-PCR samples for DNA sequencing. All samples were positive using conventional PCR, as shown in Figure-1.

### Prevalence of antimicrobial resistance genes

**Figure-2:**
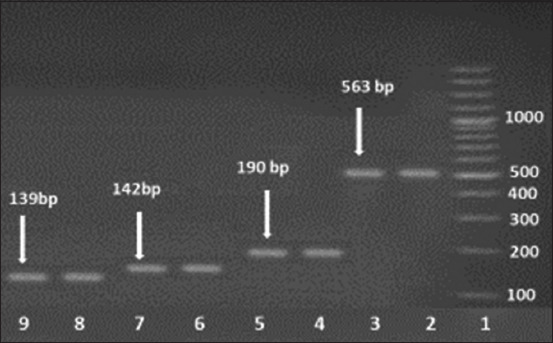
Polymerase chain reaction analysis of erythromycin-resistant determinants: Lane 1, 100 bp DNA ladder; Lanes 2 and 3, *erm*T (536 bp); Lanes 4 and 5, *erm*C (190 bp); Lanes 6 and 7, *erm*B (142 bp); Lanes 8 and 9; *erm*A (139 bp).

**Figure-3:**
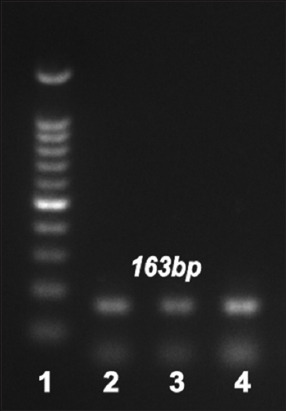
Polymerase chain reaction analysis of macrolide-resistant determinants: *msr*A (163 bp). Lane 1, 100 bp DNA ladder; Lanes, 2–4, positive samples.

Erythromycin and macrolide genes were detected in the genomic DNA of 31 *S. aureus* strains. Concerning erythromycin resistance, *erm*A-, *erm*B-, *erm*C-, and *erm*T-specific amplicons were detected in 5 (8%), 26 (41.9%), 18 (29%), and 15 (24.1%) strains, respectively, as shown in Figures-2 and Table-7. *msr*A was harbored in three (4.8%) *S. aureus* strains exhibiting macrolide resistance, as shown in Figure-3 and Table-7.

**Table-7:** Prevalence of *Staphylococcus aureus* antibiotic resistance genes (erythromycin and macrolides).

Number	Gene	Total (%)
1	*nuc*	62 (100)
2	*erm*A	5 (8)
3	*erm*B	26 (41.9)
4	*erm*C	18 (29)
5	*erm*T	15 (24.1)
6	*msrA*	3 (4.8)

### Phylogenetic analysis

**Figure-4:**
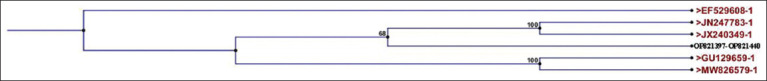
Phylogenetic relationship of selected strains of *Staphylococcus aureus* from different sources, based on the *nuc* gene.

All eight PCR products had the same nucleotide sequence and they were named OP821405–Op821412. Multiple sequence alignment, phylogenetic analysis, and tree construction of *S. aureus* *nuc* confirmed its high homology (100%) with that of *S. aureus* strains isolated from milk in bovines with mastitis in India and Kenya (JX240349, JN247783, GU129659, and MW826579), as shown in Figure-4.

### Histopathological examination

**Figure-5:**
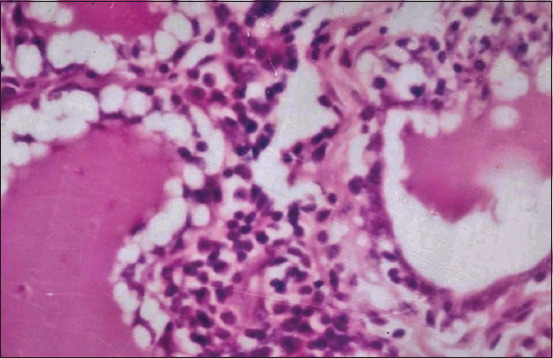
Mammary gland showing lymphocytic mastitis as diffuse infiltration of lymphocytes in the intralobular interstitium associated with homogenous esinophilic masses corpora amylacea (H and E, ×100).

Microscopic examination revealed lymphocytic mastitis in the mammary glands of the examined animals characterized by massive aggregation of mononuclear inflammatory cells, mainly lymphocytes and macrophages, in the interstitial connective tissue. The secretory acini showed vacuolar degeneration of the epithelial lining in some cases (Figure-5).

## Discussion

In Egypt and other countries, *S. aureus* is the most frequent cause of CM and SCM [1, 2, 41]. Bovine mastitis is the most common and economically significant disease in dairy animals. Among 33 bulk tank milk samples from cattle and buffaloes with SCM, *S. aureus* was isolated from 12/18 (66.7%) and 9/15 (60%) samples, respectively. In the present study, the incidence of SCM in apparently normal bulk tank milk from cattle and buffaloes from different governments in Egypt was 44% (33/75). The prevalence of SCM in milk samples collected from individual quarters using the CMT was 44% (88/200) in buffaloes and 52.1% (146/280) in cattle. In contrast, the incidence of SCM in milk samples from animals with no obvious abnormalities was 36.7% (55/150) in cattle and 31.9% (43/135) in buffaloes. Between cattle and buffaloes, there was no appreciable variation in the occurrence of SCM.

According to Hoque *et al*. [14], the proportion of riverine buffaloes with SCM was 37.6% (188/500). However, using the CMT, Abed *et al*. [31] reported that the prevalence of SCM was 46%. Our investigation detected SCM in 98/285 animals (34.4%). Many earlier investigations in Egypt and other countries supported these conclusions [42–45]. Poor housing and bedding materials, unsanitary conditions, a history of mastitis, improper milking techniques, and contaminated milking equipment can all contribute to high rates of SCM in dairy herds [46–48]. To reduce the adverse effects of SCM, dairy farms’ entire farming and housing systems and udder health management procedures should be addressed.

Bovine mastitis, a condition that significantly affects the dairy supply chain, is caused *by S. aureus*, which has been the focus of extensive research in the veterinary area [3]. According to Table-6, the prevalence of *S. aureus* in milk from animals with SCM raised by smallholders was 36.4% (20/55) in cattle and 48.8% (21/43) in buffaloes, whereas the overall prevalence of *S. aureus* in SCM milk samples was 41.8% (41/98). Hoque *et al*. [14] reported that 291 isolates were recovered from 188 SCM samples, and the prevalence of *S. aureus* was 37.4% (109/291), almost identical to our findings. Moreover, the prevalence of *S. aureus* in the milk samples obtained from individual quarters utilizing the CMT was reported as 35.9% (84/234), including rates of 36.3% (53/146) in cattle and 31% (31/88) in buffaloes. This result was much higher than the prevalence (6.5%) reported by Haltia *et al*. [49] but similar to findings in earlier research [50, 51]. Nevertheless, Rasool *et al*. [2] indicated that the prevalence of *S. aureus* in mastitis-affected buffaloes was 75%, whereas De Los Santos *et al*. [52] revealed that subclinical staphylococcal mastitis was present in most farms (82%). This prevalence resembles that revealed in a recent study by Akkou *et al*. [53]. The incidence reported by Ashfaq and Muhammad [54] was lower than that reported by Akkou *et al*. [53], which was higher than our findings in both cattle and buffaloes. *S. aureus* is spread among animals through the use of contaminated milk utensils and the hands of the milker [55]. Moreover, the keratin coating in the teat canals of healthy cows allows *S. aureus* to persist and resist phagocytosis [56, 57]. This emphasizes the importance of good hygiene and management procedures in dairy farms. Furthermore, this poses a major risk to public health because mastitic milk is typically added to bulk milk tanks, particularly in areas in which some people might consume raw milk or non-heated dairy products such as yogurt or cheese [58].

Molecular techniques such as PCR are employed to rapidly identify *S. aureus*, which is crucial for both treatment and the adoption of infection control strategies to stop the spread of sickness and outbreaks [20, 59]. Real-time PCR offers high sensitivity and specificity for the detection of mastitis-causing pathogens in bulk tank milk [21]. In this study, 62 *S. aureus* isolates were molecularly confirmed using probe-based real-time PCR. Our findings are consistent with those of Katholm *et al*. [60], who used real-time PCR to test bulk tank milk from 4258 Danish dairy herds. In their study, *Staphylococcus* spp. were found in all bulk tank milk samples, whereas Koskinen *et al*. [22] obtained false-positive results using real-time PCR to test quarter milk samples from clinically healthy cows with a low milk SCC. As with all techniques, real-time PCR requires comprehensive validation to prevent false-positive or false-negative results caused by its ability to identify low copy numbers of bacteria originating from the environment or by components in the sample that inhibit the reaction [21]. According to prior research, real-time PCR is more sensitive and specific than bacterial cultures for detecting mastitis pathogens [61, 62]. In herds with a high prevalence of contagious mastitis, dairy farmers frequently use antibiotic therapy as a crucial tool to prevent intramammary infection, particularly before calving, or to treat persistent and chronic udder infections, as well as for dry cow therapy at the end of the lactation season [63]. Because of the risk of resistance spreading to people and its impact on the effectiveness of current antimicrobial therapy procedures, excessive antimicrobial use in dairy farms has led to the emergence of resistance among several bacteria [64]. The macrolide erythromycin exhibits strong antibacterial activity against Gram-positive and Gram-negative bacteria, including *Staphylococcus*, *Streptococcus*, and *E. coli* [65, 66]. *Staphylococcus aureus* is a primary cause of mastitis, and it is often resistant to antibiotics. Rasool *et al*. [2] identified the genetic factors of erythromycin resistance in *S. aureus*, such as *erm*A, *erm*B, and *erm*C. This investigation used PCR to detect the erythromycin resistance profiles of the genomic DNA of 31 *S. aureus* isolates. As indicated in Figures-2 and Table-7, most *S. aureus* isolates tested positive for *erm*A, *erm*B, *erm*C, or *erm*T. Moreover, the existence of *msr*A was examined in *S. aureus* isolates resistant to macrolides, and this gene was found in three (4.8%) strains, as shown in Figure-3 and Table-7. Our findings are consistent with those of a study conducted in Pakistan that assessed erythromycin resistance profiles in milk samples and found that 8 (52.1%), 3 (21.4%), and 5 (35.7%) *S. aureus* isolates were positive for *erm*A, *erm*B, and *erm*C, respectively [2]. Our resistance rate was similar to that Rusenova *et al*. [67] reported in another investigation in Bulgaria, in which erythromycin resistance was identified in 7/12 (58%) samples. Additionally, our research is supported by a Chinese study that detected macrolide resistance in 34.08% of patients [68]. By contrast, Algammal *et al*. [42] conducted a study in Egypt to estimate the antimicrobial resistance profiles and determine the prevalence of *S. aureus* on farms in Ismailia Province. Their study revealed that 63.1% of the *S. aureus* strains were sensitive to erythromycin, and the different findings could be attributable to the different locations in which the studies were conducted. De Los Santos *et al*. [52] examined subclinical bovine mastitis associated with *Staphylococcus* in Uruguayan dairy farms, and their results disagreed with our findings. None of their *Staphylococcus* isolates showed resistance to erythromycin. In addition, isolates identified by Hoque *et al*. [14] were extremely susceptible to azithromycin (80.7%) and erythromycin (60.5%). Their earlier investigations and several other studies reported the highest vulnerability of mastitis-causing bacterial infections to macrolides, such as azithromycin and erythromycin [4, 69, 70].

Eight PCR products were obtained in DNA sequencing in this study using standard PCR, and multiple sequence alignment, phylogenetic analysis, and tree construction of the *S. aureus nuc* gene revealed a high degree of homology (100%) with that in *S. aureus* strains isolated from milk in bovines with mastitis in India and Kenya.

The production of different toxins, virulence factors, and cell wall adhesion proteins is most likely attributable to the ability of *S. aureus* to cause mastitis [71, 72]. The bacterium can withstand phagocytosis in the udder, and it frequently leads to persistent inflammation [71, 73]. Because changes in udder tissue occur far sooner than they become obvious, early identification of SCM is crucial [14].

Our histological analysis of udder tissues revealed significant accumulation of mononuclear inflammatory cells, primarily lymphocytes and macrophages, in the interstitial connective tissue. In some instances, the epithelial lining of the vacuoles in the secretory acini was degenerated. This finding aligns with that of Fasulkov *et al*. [74], who recorded histological findings revealing epithelial cell vacuolar degeneration, interstitial alterations, edema, and mononuclear inflammatory cell proliferation (lymphocytes and histiocytes).

## Conclusion

Our findings showed that *S. aureus* had a high prevalence in raw milk. Moreover, many erythromycin and macrolide resistance genes were present in the identified *S. aureus* isolates. Therefore, developing treatment strategies based on the various regional and seasonal characteristics linked to the occurrence and resistance of *S. aureus* strains is extremely important. Our findings will assist in safeguarding public and animal health by preventing *S. aureus* infections in cattle on dairy farms. The *S. aureus* strains isolated from submastitic milk samples could be recognized within 2 h based on the high sensitivity and specificity of probe-based real-time PCR. This approach is probably useful for the rapid screening of *S. aureus* isolates and the efficient treatment and control of *S. aureus* mastitis.

## Authors’ Contributions

KAA, EAF, and AAA: Study design, conducted the PCR, genetic markers of virulence, and antibiotic resistance. AHS: Pathological examination. ME: Diagnosis of SCM using CMT and statistical analysis. AMA: Bacterial isolation and identification and nucleic acid extraction. All authors have read, reviewed, and approved the final manuscript.
